# The Relationship Between Vitamin B6, Diabetes and Cancer

**DOI:** 10.3389/fgene.2018.00388

**Published:** 2018-09-13

**Authors:** Chiara Merigliano, Elisa Mascolo, Romina Burla, Isabella Saggio, Fiammetta Vernì

**Affiliations:** ^1^Dipartimento di Biologia e Biotecnologie “C. Darwin," Sapienza Università di Roma, Rome, Italy; ^2^Institute of Structural Biology, School of Biological Sciences, Nanyang Technological University, Singapore, Singapore

**Keywords:** vitamin B6, pyridoxal 5′- phosphate, diabetes, chromosome aberrations, *Drosophila*, AGEs

## Abstract

Pyridoxal 5′-phosphate (PLP), the active form of vitamin B6, works as cofactor in numerous enzymatic reactions and it behaves as antioxidant molecule. PLP deficiency has been associated to many human pathologies including cancer and diabetes and the mechanism behind this connection is now becoming clearer. Inadequate intake of this vitamin increases the risk of many cancers; furthermore, PLP deprivation impairs insulin secretion in rats, whereas PLP supplementation prevents diabetic complications and improves gestational diabetes. Growing evidence shows that diabetes and cancer are correlated not only because they share same risk factors but also because diabetic patients have a higher risk of developing tumors, although the underlying mechanisms remain elusive. In this review, we will explore data obtained in *Drosophila* revealing the existence of a connection between vitamin B6, DNA damage and diabetes, as flies in the past decade turned out to be a promising model also for metabolic diseases including diabetes. We will focus on recent studies that revealed a specific role for PLP in maintaining chromosome integrity and glucose homeostasis, and we will show that these aspects are correlated. In addition, we will discuss recent data identifying PLP as a putative linking factor between diabetes and cancer.

## Vitamin B6

The biologically active form of the vitamin B6, the pyridoxal 5′-phosphate (PLP), acts as coenzyme in about 160 distinct enzymatic activities mainly involved in amino acid, carbohydrate and lipid metabolism, and plays key roles in the synthesis and/or catabolism of certain neurotransmitters (Percudani and Peracchi, 2003; di Salvo et al., 2011). In addition, PLP works as antioxidant molecule by quenching oxygen reactive species (ROS) (Ehrenshaft et al., 1999) and counteracting the formation of Advanced Glycation End products (AGEs), genotoxic compounds associated with senescence and diabetes (Booth et al., 1997). Mammals, differently from microorganisms, are not able to synthesize PLP but they recycle it through a *salvage pathway* from B6 vitamers as pyridoxal (PL), pyridoxamine (PM), and pyridoxine (PN) contained in food (McCormick and Chen, 1999). In the cytoplasm PL, PM, and PN are converted into the 5′-phosphorylated vitamers by pyridoxal kinase (PDXK), while the FMN-dependent pyridoxine 5′-phosphate oxidase (PNPO) converts PNP and PMP into PLP.

Deficiency of vitamin B6 has been implicated in several clinically relevant diseases including autism, schizophrenia, Alzheimer, Parkinson, epilepsy, Down’s syndrome, diabetes, and cancer. In this review we focus on the role of PLP in diabetes and cancer suggesting more specialist readings for the other pathologies (Hellmann and Mooney, 2010; di Salvo et al., 2012).

## Vitamin B6 in Cancer and Diabetes

Epidemiological studies and meta-analysis indicate an inverse correlation between vitamin B6 and cancer development. For example, high expression levels of PDXK have been positively correlated with survival of non-small cell lung cancer (NSCLC) patients (Galluzzi et al., 2012). Furthermore, vitamin B6 intake and blood PLP levels were inversely correlated with the colorectal cancer risk (Gylling et al., 2017). PLP has been proposed to influence carcinogenesis through different pathways including those involved in DNA metabolism, suggesting that antitumor properties of vitamin B6 may be in part due to its protective role against DNA damage (Ames and Wakimoto, 2002). Vitamin B6 has also been associated to diabetes. However, it is not clear whether low PLP levels represent a cause or an effect of diabetes or both. Some studies report that low PLP levels can contribute to cause diabetes (Toyota et al., 1981; Rubi, 2012), whereas others show that diabetes decreases PLP levels (Bennink and Schreurs, 1975; Spellacy et al., 1977; Okada et al., 1999). Several groups reported that B6 administration produces beneficial effects on diabetic pathology and its complications (Cohen et al., 1984; Solomon and Cohen, 1989; Ellis et al., 1991; Hayakawa and Shibata, 1991; Jain, 2007), although underlying cellular and molecular mechanisms are not completely understood.

Pyridoxal 5′-phosphate deficiency might impact on diabetes in different ways. For example, it could act on the pathway that converts tryptophan into nicotinic acid as PLP is a cofactor of some enzymes that work in this pathway (Bennink and Schreurs, 1975; Spellacy et al., 1977; Oxenkrug, 2013). It has been shown that metabolites produced when this pathway does not work properly can interfere with biological insulin activity (Kotake et al., 1975) causing insulin resistance, a hallmark of type 2 diabetes. Moreover, it has also been proposed that PLP may impact on insulin resistance by controlling the expression of genes involved in adipogenesis (Moreno-Navarrete et al., 2016). Another hypothesis is that PLP deficiency might cause insulin resistance through an increase of homocysteine due to impairment of enzymes such as cystathionine-β-synthase (CBS) and cystathionine-γ-lyase (CGL), which require PLP as a coenzyme (Liu et al., 2016).

Cancer and diabetes are correlated as they share some risk factors. Growing evidence shows that diabetic patients have an increased risk to develop some malignance throughout multiple, not fully elucidate mechanisms, including DNA damage (Noto et al., 2011; Dankner et al., 2016). Interpret the cause-effect relationships in humans is difficult for unavailability of controls and high costs of human research. Researches in the field thus rely on model organisms as well as human 3D cultures and stem cell based systems (Riminucci et al., 2006; Simao et al., 2016). In this review we show how *Drosophila* has turned out to be a useful model not only to investigate the role of vitamin B6 in cancer and in diabetes but also to connect these two pathologies. Furthermore, we present evidence from flies suggesting that incorrect PLP intake could represent a cancer risk factor for diabetic patients, as it enhances DNA damage.

## PLP Safeguards Genome Integrity in *Drosophila*

Using *Drosophila* as a model system we demonstrated that PLP plays a crucial role in genome integrity maintenance (Marzio et al., 2014). *Drosophila dPdxk* gene encodes the ortholog of the PDXK enzyme, which is required for vitamin B6 biosynthesis. Mutations in *dPdxk* gene produce, in larval neuroblasts, chromosome aberrations (CABs) (∼6 vs. 0.5% in controls), which are fully rescued by PLP. Dividing larval neuroblasts represent a suitable system to study CABs in *Drosophila* as they exhibit morphologically well defined chromosomes that can be stained by a variety of procedures (Gatti and Goldberg, 1991). CABs have been previously observed after X-ray treatment (Gatti et al., 1974) and as a consequence of mutations in genes which control chromatin structure (Mengoli et al., 2014) or different steps of DNA repair (Bianchi et al., 2017; Merigliano et al., 2017). Vitamin B6 antagonists, namely 4-deoxypyridoxine hydrochloride (4-DP), penicillamine, cycloserine, or isoniazid, produce high CAB frequencies (ranging from 3 to 19%) in wild type cells, further confirming that PLP plays an essential role in genome integrity maintenance. The aforementioned function is evolutionarily conserved in humans as the depletion of the human *PDXK* counterpart induces CABs and a copy of the human *PDXK* gene inserted in a *Drosophila dPdxk^*1*^* background is capable to rescue CABs (Marzio et al., 2014). Also in *Saccharomyces cerevisiae* mutations in *BUD16* gene, encoding PDXK, result in gross chromosomal rearrangements. Altogether these data support the hypothesis that low PLP levels may promote cancer initiation and progression throughout the formation of CABs, which represent a cancer prerequisite (Mitelman et al., 2007; Aguilera and Gomez-Gonzalez, 2008; Bunting and Nussenzweig, 2013).

## DNA Damage is Caused by High Glucose Levels in PLP Deficient Cells

We obtained evidence that in *Drosophila* PLP is involved in glucose metabolism as *dPdxk^*1*^* mutants display, in their hemolymph, higher glucose concentrations compared to wild type individuals. *dPdxk^*1*^* mutants have normal insulin levels, but a weakened ability to respond to insulin signaling (Marzio et al., 2014). Remarkably, in *dPdxk^*1*^* mutants, high glucose levels and CABs are correlated. In *dPdxk^*1*^* mutant brains, indeed, 1% glucose *in vitro* treatment strongly increases CAB frequency (from 6 to 20%); in contrast sugar treatment of wild type larvae and brains did not result in detectable effects on chromosome integrity. The relationship between glucose and CABs, in PLP depleted cells, is evolutionarily conserved as glucose supplementation enhances chromosome damage also in *PDXK* depleted HeLa cells (Marzio et al., 2014). In addition, the wild type *PDXK* human gene, inserted in *dPdxk^*1*^* flies, is able to reduce hyperglycemia. Hyperglycemia triggers the formation of AGEs that in turn produces ROS, which are harmful for DNA. It has been shown that ROS, even at low levels, can cause DNA damage that further leads to DNA double strands breaks (DSBs) (Sharma et al., 2016) and that CABs are mainly generated by unrepaired or improperly repaired DSBs. Repairing complex DSBs may result in genomic instability that can be involved in the etiology of a wide variety of human diseases including cancer (Khanna and Jackson, 2001; Kasparek and Humphrey, 2011).

*dPdxk^*1*^* mutant cells accumulate AGEs and treatment of *dPdxk^*1*^* mutants with alpha lipoic acid (ALA), a known AGE inhibitor, rescues not only AGEs but also CABs, suggesting that PLP protects from DNA damage *Drosophila* cells by counteracting AGE formation (Marzio et al., 2014). To the best of our knowledge only our work (Marzio et al., 2014) showed the cause effect relationship between AGEs and CABs in flies. However, *Drosophila* represents a good model to study AGEs as flies accumulate significant AGEs over their lifespan (Oudes et al., 1998) and, in addition, an AGE-rich diet results in ROS accumulation (Tsakiri et al., 2013). AGE formation is at the basis of many diabetic complications (Thorpe and Baynes, 1996; Brownlee, 2001; Vlassara and Palace, 2002) and it also can contribute to diabetes onset (Vlassara and Uribarri, 2014). Our data are consistent with studies indicating that vitamin B6 is beneficial for diabetes complication as, for example, nephropathy (Hayakawa and Shibata, 1991) and retinopathy (Ellis et al., 1991) and with *in vivo* studies showing that PLP is able to reduce AGE accumulation and protein glycation (Cohen et al., 1984; Solomon and Cohen, 1989). How PLP counteracts AGEs is not completely understood but it has been proposed that it may trap 3 deoxyglucosone (3-DG), an AGE’s metabolism intermediate (Nakamura et al., 2007), although other mechanisms are possible. Besides to its antioxidant role, PLP also works as cofactor for serine hydroxymethyltransferase enzyme, which takes part to the thymidylate synthase cycle by converting dUMP in dTMP (Florio et al., 2011). However, whereas PLP depletion in yeast compromises DNA synthesis (Kanellis et al., 2007), in *Drosophila* it does not seems to have the same effect. Although in *dPdxk^*1*^* mutants there is an altered dTMP/dUTP ratio DNA syntesis is not the main cause of CABs as *dPdxk^*1*^* mutant are only slighty sensitive to Hydroxyurea, a drug that interferes with replication (Marzio et al., 2014). However, considering the wide range of enzymatic reactions regulated by vitamin B6, we cannot exclude that in addition to block AGE formation PLP may prevent CABs also through other mechanisms.

## *Drosophila* as Type 2 Diabetes Model

*Drosophila* represents a good model to study diabetes as flies and humans largely share mechanisms involved in glucose homeostasis maintenance (Graham and Pick, 2017). In addition, fly genome possess well characterized orthologs of most genes working in the insulin signaling pathway that controls the glucose uptake and storage (Garofalo, 2002).

In humans and mice mutations in insulin pathway genes cause severe insulin resistance syndromes and type 2 diabetes (reviewed in Boucher et al., 2014). In *Drosophila* type 2 diabetes models can be generated by two different strategies: by downregulating conserved genes working on insulin pathways as for example the insulin receptor *InR*, the insulin substrate receptor *chico/IRS1, Akt1, PI3K*, and by feeding flies with a high sugar rich diet (Alfa and Kim, 2016). In both cases resulting flies exhibit diabetic hallmarks as hyperglycemia and insulin resistance allowing the study in flies of various aspects of diabetes and related human disorders. In addition, a diabetic fly model also enhances the ability to identify genes and discover functional interactions that can be exploited for disease treatment.

## PLP Depletion as New Cancer Risk Factor in Diabetic Cells

Meta-analysis and epidemiological studies indicate that diabetic patients have an increased risk to develop several solid and hematologic malignancies (including liver, pancreas, colorectal, kidney, bladder, endometrial, and breast cancers, and non-Hodgkin’s lymphoma) although the molecular mechanisms are not completely clarified (Vigneri, 2009; Noto et al., 2011; Dankner et al., 2016). However, some risk factors have been identified including hyperinsulinemia and hyperglycemia that might rise cancer risk in diabetic patients by promoting cell growth (Shikata et al., 2013). Besides triggering cell division hyperglycemia also causes oxidative stress as glucose in excess promotes, through different pathways, ROS formation which in turn induces DNA and cellular damage (Rains and Jain, 2011). In addition, in cells from diabetic patients an impaired DNA repair, combined to a weakened antioxidant defense, contributes to enhance DNA damage (Blasiak et al., 2004). Consistently, oxidative damage and DNA strand breaks have been found in both type 1 and type 2 diabetic patients (Goodarzi et al., 2010; Tatsch et al., 2012; Anand et al., 2014). We have recently shown in *Drosophila* that PLP deficiency can further increase DNA damage in cells from diabetic individuals (Merigliano et al., 2018). Using two different type 2 diabetes models, the first obtained by downregulating genes involved in insulin signaling such as *InR*, *chico (IRS1)*, and *Akt1*, and the second by feeding wild type flies with a high sugar diet (Musselman et al., 2011), we showed that the treatment of larval neuroblasts with the strong PLP inhibitor 4-DP produced a very high CAB frequency ranging from 60 to 80% (vs. 25% in wild type cells). Accordingly, genetic analysis revealed a synergistic interaction between *Akt1* and *dPdkx^*1*^* mutations in CAB formation (Merigliano et al., 2018). AGEs are in part responsible for CABs in *Drosophila* diabetic PLP depleted cells as they accumulate in these cells and, more strikingly, ALA rescues either AGEs and CABs (Merigliano et al., 2018). These findings indicate that, in diabetic cells, low PLP levels heavily impact on genome integrity. Thus, if translated to humans, these data suggest that low PLP levels may contribute to increase cancer risk in diabetic patients. Although PLP deficiency is a rare condition caused by excessive alcohol consumption, unwanted effects of some drugs (i.e., isonyazide, cycloserine penicillamine), or celiac disease and renal dialysis (Clayton, 2006), it has been demonstrated, in murine models and by epidemiological studies, that it can also be associated to diabetes (Leklem and Hollenbeck, 1990; Okada et al., 1999; Ahn et al., 2011; Nix et al., 2015). All evidence suggests the importance to maintain under strict control PLP levels in diabetic patients to avoid the chance to increase DNA damage, which could in turn contribute to cancer initiation and progression.

## Conclusion

Several studies have shown that insufficient intake of vitamin B6 is associated with increased cancer risk and growing evidence indicates that diabetes patients have a higher risk of developing various types of cancer. The findings reviewed here, obtained in *Drosophila*, provide a mechanistic link between aforementioned studies by suggesting that PLP deficiency accompanied by hyperglycemia can lead to DNA damage and may contribute to cancerogenesis. Thus, *Drosophila* has proved to be a useful model system to shed light on a novel and important role of vitamin B6 deficiency in the pathogenesis of cancer and diabetes. In addition, this model organism allowed identifying PLP deficiency as one of the risk factors that contribute to correlating diabetes to cancer.

## Author Contributions

CM contributed to planning and writing the manuscript. EM and RB contributed to planning the study. IS contributed to writing the manuscript. FV planned the study and wrote the manuscript. All authors performed a critical revision of the manuscript.

## Conflict of Interest Statement

The authors declare that the research was conducted in the absence of any commercial or financial relationships that could be construed as a potential conflict of interest. The reviewer RC declared a shared affiliation, with no collaboration, with the authors to the handling Editor at time of review.
